# Reliability and validity of Azeri Turkish version of geriatric depression scale

**DOI:** 10.15171/hpp.2020.12

**Published:** 2020-01-28

**Authors:** Sepideh Herizchi, Habibeh Barzegar, Shahrokh Amiri, Ali Fakhari, Homayoun Sadeghi-Bazargani, Seyed Gholamreza Noorazar, Mostafa Farahbakhsh, Mostafa Ghaneei

**Affiliations:** ^1^Research Center of Psychiatry and Behavioral Sciences, Tabriz University of Medical Sciences, Tabriz, Iran; ^2^Road Traffic Injury Research Center, Tabriz University of Medical Sciences, Tabriz, Iran; ^3^Tabriz Health Services Management Research Center, Tabriz University of Medical Sciences, Tabriz, Iran

**Keywords:** Depression, Older adults, Geriatric depression scale, Reliability, Validity

## Abstract

**Background:** In older adults, depression symptoms may be masked by physical complaints and be even attributed to the natural aging process, which may be resulted in improper diagnosis. Native-language scales can be highly effective in the detection of depressive disorders. In this study we attempted to assess the reliability and validity of the Azeri Turkish version of the geriatric depression scale (GDS).

**Methods:** In this psychometric study, the GDS and the Structured Clinical Interview for DSM IV(SCID) questionnaires were administered to a sample of 387 older adults (60 years and older)from the member households of Tabriz health centers. The English version of GDS was translated into Azeri Turkish. Translation-back translation process was conducted. The receiver operating characteristics (ROC) curve, as well as sensitivity and specificity, were used to determine the validity of the questionnaire, and the test-retest method was used to calculate reliability.

**Results:** The mean age of participants was 69.30. The area under the ROC curve for the scores higher than five was 0.832 and for the scores equal to ten and above was 0.871. The sensitivity and specificity for the scores higher than five were 90.9% and 73.4%, respectively. The reliability of this scale was confirmed based on intraclass coefficient (ICC) = 0.79.

**Conclusion** : The Azeri Turkish version of GDS was found with appropriate levels of validity and reliability.

## Introduction

The world’s population is aging. It is anticipated that by the year 2025 two thirds of people older than 65 will be living in developing countries.^1^ Among all types of physical and mental disorders in the elderly, depression is one of the most common disorders, which may increase significantly during hospital admissions due to serious physical illnesses and decreased cognitive and physical functions (features of old age).^2-5^ This disease affects the quality of life (QOL) of older adults. Depression symptoms may be masked by physical complaints and be even attributed to the natural aging process, which may be resulted in improper diagnosis. Late-life depression (LLD) is a serious challenge and one of the most common psychiatric disorders due to the increased aging population. Several previous studies consider LLD as a major depressive disorder occurring for the first time in people aged 60 or over.^6-9^ Depressive disorders are seen in 17%-37% of older adults attending primary care centers, 30% of whom are detected to be suffering from a major depressive disorder.

Many assessment instruments have been developed to assess LLD in developed countries. Among the most effective instruments, geriatric depression scale (GDS) is a frequently used scale in clinical trials and screenings for depressive disorder in the elderly populations. This scale is a self-report instrument with a “yes-no” simple response style, which makes it appropriate to be used for people with cognitive impairments.^10^

Psychometric evaluation of questionnaires among older populations may be very difficult, due to age-related and cognitive impairments and also the lack of specific psychological assessment scales for the elderly.^11^ In previous studies, test-retest reliability and internal consistency of the GDS scale were reported to be in the ranges of 0.85-0.90 and 0.89-0.94, respectively.^12,13^ Iran is a country with several ethnic groups and various local languages. So, a majority of elderly people are not fluent in Persian. Also, due to poor literacy of older adults in Iran there is a need for the translated versions of such psychometric assessment instruments into the local languages. In the present study, we sought to prepare the Azeri Turkish version of the GDS due to the large number of Turkish-speaking people within the country.

## Materials and Methods

### Participants and procedures

This research was a cross-sectional study conducted in Tabriz during 12 months in 2018. The cluster sampling method was used to recruit the respondents. To calculate the number of clusters, the health care complex assessment method described in Tabrizi et al was used.^14^ Among all twenty-household clusters, 150 clusters were randomly selected based on the population census results in the healthcare centers and the clusters selected in previous health surveys (demographic surveys, STEPS survey).^14^ All older adults in these clusters were included in the study. The term “elderly” was used in this study to refer to people aged 60 years and older. This study is part of a megaproject called the Mental Health Assessment in the Elderly that are conducted in Tabriz and supported by the Research Center of Psychiatry and Behavioral Sciences. The sample size in this megaproject was estimated to be 1120 older adults based on the Cochran sampling formula with the precision (d) of 0.025, variance (pq) of 0.9, and confidence level of 95%.

Considering the effect of the sampling plan (DE) and the general plan of the Elderly Mental Health Project, a total sample of 1000 respondents were questioned out of which 387 people were entered into the project. Four participants were excluded from the analysis due to incomplete information. The final sample size was 383. The inclusion criteria for the participants were as follow: Mastery of Azeri Turkish, no cognitive impairment, over 60 years old, and no history of psychiatric disorders.

### Measurements

*Demographics questionnaire*

This questionnaire contained questions about variables such as age, gender, retirement, and age in retirement.

*Persian version of GDS*

This scale is known as the most effective instrument for LLD screening. It is a self-report test with 15 questions. It was developed by Yesavage et al in 1982 to measure LLD. This questionnaire has two subscales including depression and psychosocial activities.^12^ Its Persian version was previously validated by Malakouti et al. The results of the tests showed good reliability coefficients. Its Cronbach’s alpha was 0.9, and its sensitivity and specificity were 0.9 and 0.84, respectively.^15^

*Six-item screener (SIS) measurement scale*

This tool has been used in previous studies to screen for cognitive impairments. This scale was used to exclude the cognitively impaired individuals from the study. The SIS includes 6 explicit items (year, month, time, and clock drawing, etc). In a previous study Xue et al reported the Cronbach’s alpha of the scale to be 0.7.Compared to the MMSE scale, its sensitivity and specificity were 0.86 and 0.87, respectively.^16^ The questionnaire was translated into Persian by the researchers and was provided to five psychiatrists to determine face validity. Based on their suggestions, a final questionnaire was prepared and used. SIS was completed firstly to find any cognitive impairment among participants prior to providing them with the other questionnaires.

*SCID questionnaire*

The structured clinical interview is based on DSM-TV-TR criteria and has two clinical and research versions. Both versions have self-report questionnaires for assessing psychiatric disorders and personality disorders. This form is the most widely used diagnostic interview instrument in psychiatric trials throughout the world. Previous studies have reported the reliability (Overall weighted kappa was 0.52 for current diagnoses and 0.55 for lifetime diagnoses) and validity (specificity >0.85) of the Persian version of SCID-IV for use in clinical and research environments.^17,18^ The clinical version of the SCID-IV was used in this study to detect psychiatric disorders. A set of standardized items are provided for each disorder based on DSM-IV in this questionnaire, which comprise all psychiatric disorders.

### Psychometric stages

**Figure 1:**
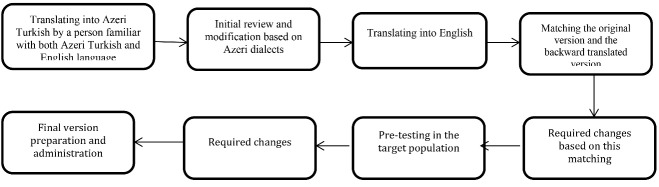
Diagram of study protocol.

This research was designed to translate and localize and evaluate the psychometric properties of the GDS. The study was conducted in three stages (Figure 1).

*Stage I: Translating the questionnaire into Turkish*

At this stage, the original English version of the 15-item GDS was translated into Azeri Turkish in accordance with the translation protocol of the International Quality of Life Assessment (IQOLA) project.^19^ For this purpose, a translator whose native language was Azeri Turkish and was experienced enough in translating English texts translated the English version of the questionnaire into Turkish. The translator was also asked to provide a list of possible alternative translations for some of the words, phrases, and sentences of the questionnaire, if necessary. The prepared Turkish version was presented to a person professional with Turkish and English to be back-translated into the original English language. Then, the back-translated and the original versions were presented to two professional English interlocutors in order for them to determine the extent to which the two questionnaires matched based on a checklist. Subsequently, the poorly matching questions were back-translated and the final version was prepared after the required amendments. The Turkish version of the questionnaire was also presented to ten people professional with the Azeri Turkish language in order for them to modify the items based on the colloquial language. The scale was also completed by ten older adults as a pilot test, and their comments were used in preparation of the final version. Two specialists with a master’s degree in clinical psychology and work experience of five years were selected and trained on how the scale should be completed. Finally, the questionnaire was read aloud to them. Then, they were asked to read it to ensure they would not have any difficulty in conveying the items to the selected sample.

*Stage II: Validity assessment*

*Face validity*

To determine face validity of the questionnaire, we prepared a form in which the transparency of sentences and suitability with the cultural conditions were measured on a four-point scale. The questionnaire was sent to ten experts in psychology. The items scored less than 80% were back-translated, and modified based on the experts’ opinions.

*Criterion validity*

To determine construct validity, the Persian version of the questionnaire was also used simultaneously. The Persian version was initially completed to prevent the effect of questionnaire completion order. To determine validity of the questionnaire, 387 older adults selected in the megaproject were interviewed with the Azeri Turkish version of the GDS.

*Stage III: Reliability assessment*

To assess the reliability of the questionnaire, 20 older adults completed the questionnaire twice at a one-week interval.

### Statistical analysis

In the statistical analysis, we aimed to compare the ability of the GDS in distinguishing between the older adults with and without depression. The underlying and demographic variables were analyzed using measures of central indicators and dispersion such as mean, standard deviation, and frequency. The data were analyzed using the IBM SPSS version 23. To determine reliability, the test-retest method was conducted with Kendall test. Also, criterion validity was used to determine validity, and sensitivity and specificity indicators, as well. In order to calculate the sensitivity and specificity, the psychiatric interview by SCID was considered as the main criterion. Then, the results obtained from data analysis were compared to them and sensitivity and specificity were calculated using the ROC curve test.

## Results

The participants consisted of 383 older adults with the mean age of 69.30 (SD 7.50). About 47% (182) were male with the mean age of 70.27 (SD 7.73), and 52% (201) were female with the mean age of 68.43 (SD 7.53). In terms of education level, the highest and the lowest frequencies were for “secondary and high school” (35.2%) and “academic education” (10%), respectively. Table 1 presents the percentage of participants in terms of literacy and gender.

The ROC curve for the GDS test scores greater than or equal to five was calculated and plotted with 383 acceptable cases. The area under the curve (AUC) was 0.832 (95% CI: 0.79-0.89) (Table 2). ROC curve for the GDS test scores greater than or equal to 10 was also calculated and plotted with 383 acceptable cases (Table 3). The AUC was 0.871(95% CI: 0.82-0.91). It should be noted that the ROC curve is plotted based on the sensitivity and 1- specificity scales. In this study, sensitivity shows the likelihood of a positive diagnosis of depression by GDS in people diagnosed with depression based on the SCID scale; specificity shows the likelihood of a negative diagnosis of depression by GDS in people diagnosed without depression based on the SCID scale. The ROC curve also shows the trade-off between these two scales. As this score was higher in the scores above 10, compared to the scores above 5, we will rely on the results of the scores above 10 (Table 4).

The prevalence of depression based on the SCID scale was 11.4% in the total population (Table 5). The prevalence of depression in the GDS was 32% (95% CI: 27.3-36.7) for the scores above 5 and 18.3% (95% CI: 14.5-22.2) for the scores equal to or greater than 10, which is closer to the prevalence rate obtained from the SCID scales. The reliability of the scale was 67.3% based on test –retest Kendall method. Intraclass correlation coefficient (ICC) was 0.79.

## Discussion

As a widely recommended screening tool for LLD, the GDS-15 is very useful in diagnosing major depression at old ages in healthcare systems. Using the SCID scale and the Azeri Turkish version of GDS, the prevalence of depression was obtained 11.4% and 18.3%, respectively. These findings are consistent with the results of a meta-analysis of 74 studies on the prevalence of depression in the world, showing the prevalence rate of 10.3% (between 4.7% and 16%).^20^

In the present study, the AUC for prediction and diagnostic power was 0.871, which is consistent with the results reported in previous studies. One^21^ of these studies investigated the psychometric properties of the 15-item GDS in a sample of 960 patients aged 65 and over with functional and cognitive impairments in several states of the United States. In the study, the 15-item GDS and the short form of neuro-psychiatric interview were used to measure depression, life satisfaction, suicidal thoughts, and suicide attempts. The factor analysis of GDS in the study showed a two-factor structure that can include two subscales of depression and a positive mood. The results of the study showed an acceptable Cronbach’s alpha coefficient and the internal consistency coefficient for the GDS. The significant relationship between GDS-15 and depression, life satisfaction, suicidal thoughts, and suicide attempts confirmed the construct validity of the instrument and good sensitivity and specificity indicators for differentiation between depressed and non-depressed individuals. In general, this study provided evidence for the acceptable psychometric properties of this instrument.

Another study in 2018^22^ conducted to evaluate the psychometric properties of the Arabic version of 30-item GDS showed a positive significant relationship between all items of this scale and the main scale. Cronbach’s alpha coefficient was 0.90 indicating a high internal consistency for the scale. So, the scale was presented as an appropriate instrument for LLD detection. Marc et al^23^ evaluated the screening ability of the GDS in the US elder home care system. Like our study, it used the SCID scale as the parallel diagnostic tool and utilized the ROC curve to compare the diagnostic powers of SCID and GDS for the sensitivity and specificity indicators. Compared to the SCID, the sensitivity of 71.8%, specificity of 78.2%, and the AUC of 0.793 were obtained for the GDS with a cut-off point of 5, showing acceptable validity for this diagnostic tool. These results were consistent with those of our study. Besides, the GDS precision was not affected by the degree of medical problems, age, and other demographic and social characteristics of individuals (even those with severe illnesses and disabilities). Galeoto et al assessed the psychometric properties of the Italian version of the GDS and showed that Cronbach’s alpha of GDS-IT for depressed subjects was 84%, test-retest reliability 91%, and concurrent validity 83%. Factor analysis also included five structural factors. These results indicated that GDS is a valid, reliable, and useful test for measuring LLD. These differences in the findings of various studies can stem from cultural differences and ethnic beliefs.

Having different dialects (Azeri Turkish) and not using Turkish as an academic language in research projects can be potential biases for this study. This research project focused only on the elderly in Tabriz population and future studies can be done in a wider sample with different dialects of Turkish language.

## Conclusion

Since the AUC obtained for the Azeri Turkish version of GDS was at an acceptable level, it can be concluded that this scale is a suitable and reliable instrument for screening of LLD and facilitating the detection of depression in Azeri-speaking older adults.

## Ethical approval

People with depressive disorder received appropriate treatment if needed. Informed consent was obtained from all participants. They were also assured that their data will never be used anywhere except for research purposes. The data were anonymous and the participants were free to withdraw from the study at any time they wished. Finally, this study was approved by the Ethics Committee in Tabriz University of Medical Sciences. (Ethics No. tbzmed.rec.1396.987).

## Competing interests

The authors declare that they have no competing interests.

## Funding

This study was conducted with financial support from the Research Center of Psychiatry and Behavioral Sciences, Tabriz University of Medical Sciences.

## Authors’ contributions

AF, SA, MF, SH, HSB and MG were involved in designing the study. MG and MF contributed to the data collection. MF and HB contributed to data analysis and preparing of the manuscript draft. All authors participated in manuscript revising and finalizing.

## Acknowledgments

This article was derived from the psychiatry specialty degree thesis of Mostafa Ghaneei at Tabriz University of Medical Sciences, Iran. The authors wish to thank Dr Alireza Nadim, Ebrahim Zal, Hassan Samoudi for scale translation and Salman Abdi, Mrs Nazmpour, Mrs Fakhimi for data collection and elderly population interviews.

**Table 1:** Level of education by gender

**Level of education**	**Men**		**Women**		**Total (%)**
	**No.**	**%**	**No.**	**%**	
Illiterate	39	22.15	64	32.4	103 (26.89)
Elementary	66	37.5	69	35	135 (35.24)
Secondary/high school	50	28.4	47	23.9	97 (25.32)
Academic	21	11.95	17	8.7	38 (9.92)

**Table 2:** Comparison of GDS screening in score 5 with SCID

	**GDS**	**>5**	**≤5**	**Total**
SCID	**+**	40	4	44
	**-**	84	259	343
	Total	124	263	387

GDS: geriatric depression scale; SCID: Structured Clinical Interview for DSM-IV.

**Table 3:** Comparison of GDS screening in score 10 with SCID

	**GDS**	**>10**	**≤10**	**Total**
SCID	**+**	37	7	44
	**-**	34	309	343
	Total	71	316	387

GDS: geriatric depression scale; SCID: Structured Clinical Interview for DSM-IV.

**Table 4:** Validity criteria according to cutoff points of >5 and ≥10 of GDS

**Validity measures**		**Amount**	**95% CI**
GDS scores above 5	Sensitivity	90.9	78.8-96.4
	Specificity	73.4	70.7-79.7
	PPV	32.25	24.15-41.24
	NPV	98.05	96.15-99.58
	Positive likelihood ratio	3.7	3.01-4.57
	Negative likelihood ratio	0.12	0.047-0.3
GDS scores ≥ 10	Sensitivity	84.1	56.2-92.07
	Specificity	90.1	68.47-92.82
	PPV	52.1	39.92-64.12
	NPV	97.8	95.49-99.1
	Positive likelihood ratio	8.48	6.01-11.96
	Negative likelihood ratio	0.18	0.089-0.349

GDS: geriatric depression scale; SCID: Structured Clinical Interview for DSM-IV; PPV: positive predictive values; NPV: negative predictive values.

**Table 5:** Prevalence of depression using the SCID, GDS at areas 5 and 10

**MDD**	**No.**	**%**
Negative with SCID	344	88.6
Positive with SCID	44	11.4
Negative with GDS at the area of 5	263	68
Positive with GDS at the area of 5	124	32
Negative with GDS at the area of 10	316	81.7
Positive with GDS at the area of 10	71	18.3

GDS: geriatric depression scale; SCID: Structured Clinical Interview for DSM-IV; MDD: Major Depression Disorder.
